# Post-transcriptional gene regulation by RNA-binding proteins in vascular endothelial dysfunction

**DOI:** 10.1007/s11427-014-4703-5

**Published:** 2014-08-08

**Authors:** HongBo Xin, KeYu Deng, MinGui Fu

**Affiliations:** Institute of Translational Medicine, Nanchang University, Nanchang, 330031 China

**Keywords:** endothelial dysfunction, vascular inflammation, RNA-binding proteins, microRNAs, post-transcriptional gene regulation

## Abstract

Endothelial cell dysfunction is a term which implies the dysregulation of normal endothelial cell functions, including impairment of the barrier functions, control of vascular tone, disturbance of proliferative and migratory capacity of endothelial cells, as well as control of leukocyte trafficking. Endothelial dysfunction is an early step in vascular inflammatory diseases such as atherosclerosis, diabetic vascular complications, sepsis-induced or severe virus infection-induced organ injuries. The expressions of inflammatory cytokines and vascular adhesion molecules induced by various stimuli, such as modified lipids, smoking, advanced glycation end products and bacteria toxin, significantly contribute to the development of endothelial dysfunction. The transcriptional regulation of inflammatory cytokines and vascular adhesion molecules has been well-studied. However, the regulation of those gene expressions at post-transcriptional level is emerging. RNA-binding proteins have emerged as critical regulators of gene expression acting predominantly at the post-transcriptional level in microRNA-dependent or independent manners. This review summarizes the latest insights into the roles of RNA-binding proteins in controlling vascular endothelial cell functions and their contribution to the pathogenesis of vascular inflammatory diseases.

The endothelium is the monolayer of endothelial cells (ECs) lining the lumen of blood vessels in every organ system. These cells form a protective barrier between all tissues and the circulating blood. Normal EC function is critical for all aspects of vascular homeostasis (i.e., control of blood vessel development, growth and differentiation; control of leukocyte trafficking; control of vascular tone; control of vascular barrier; control of platelet function, coagulation and fibrinolysis) [1–3]. EC dysfunction disrupts the balance between vasoconstriction and vasodilation and initiates a number of events that trigger EC activation and predispose the vessel wall to increased endothelial permeability, leukocyte adherence, endothelial proliferation, pro-oxidation and thrombosis. Endothelial dysfunction has been implicated in several diseases including atherosclerosis, diabetes, tumor metastasis, sepsis and severe virus infectious diseases [4–6]. Importantly, the expressions of inflammatory cytokines and vascular adhesion molecules are significantly involved in the processes of EC activation, which is regulated by the transcriptional regulation programs, as well as post-transcriptional and post-translational modifications that fine-tune this response [7,8]. microRNA (miRNA) is a key transcriptional regulator. In this regard, miRNAs have emerged as critical regulators of gene expression, acting predominantly at the post-transcriptional level [9–11]. RNA-binding proteins have also emerged as critical regulators of gene expression at the post-transcriptional level. They can act in a miRNA-dependent or independent way.

Since the functions of miRNAs in vascular endothelial dysfunction have been comprehensively reviewed elsewhere [12–15], in the present review we summarize the latest insights into the roles of RNA-binding proteins in controlling vascular endothelial cell functions and their contributions to the pathogenesis of vascular inflammatory diseases.

## 1 Endothelial dysfunction in human diseases

The endothelium maintains normal vascular homeostasis with no or little expression of proinflammatory factors under normal homeostatic conditions. However, both traditional and novel cardiovascular risk factors including smoking, aging, hypercholesterolemia, hypertension, hyperglycemia, and a family history of premature atherosclerotic disease are associated with alteration in endothelial function [4–6]. In addition, infections with bacteria and viruses also impair the blood-tissue barrier and result in tissue/organ injuries [16,17]. This results in a chronic or acute inflammatory process accompanied by loss of antithrombotic factors and an increase in proinflammatory cytokines and prothrombotic products, in addition to abnormal vasoreactivity, therefore elevating risk of cardiovascular events and organ injuries.

### 1.1 Endothelial dysfunction and atherosclerosis

Atherosclerosis is characterized by the thickening of the arterial wall and is the primary cause of coronary artery disease and cerebrovascular disease, two of the most common causes of illness and death worldwide [18]. A crucial step in atherogenesis is the arterial recruitment of inflammatory cells from the circulation and their trans-endothelial migration into the sub-endothelial space of large arteries where they differentiate into macrophages and become functionally active. The endothelial activation is the first step in the development of atherosclerosis [19]. In response to inflammatory stimuli, such as tumor necrosis factor α (TNFα), interleukin 1β (IL-1β), and interferon gamma, ECs undergo inflammatory activation, resulting in an increased surface expression of cell adhesion molecules, such as vascular cell adhesion molecule (VCAM)-1, intercellular adhesion molecule (ICAM)-1, and E-selectin, which contributes to the recruitment of inflammatory cells to arterial wall and their transmigration across the wall. The activated ECs also release cytokines and chemokines such as monocyte chemotactic protein 1 (MCP-1), which is a potent inducer for monocyte attachment to ECs and migration into subendothelial space. Endothelial activation represents a switch from a quiescent phenotype toward a proinflammatory and prothrombotic phenotype. Indeed, most cardiovascular risk factors induce the expression of chemokines, cytokines, and adhesion molecules designed to interact with leukocytes and platelets in endothelium [20,21]. The molecular regulation of the gene expression program in endothelium is an intensively studied area.

### 1.2 Endothelial dysfunction and metabolic syndromes

Metabolic syndrome is a cluster of metabolic abnormalities that includes visceral obesity, dyslipidemia, hypertension, and impairment of glucose metabolism, which has been related to both endothelial dysfunction and increased risk for cardiovascular diseases and type 2 diabetes. Insulin resistance has been recognized as a common cause for clustering of these risk factors, whereas endothelial dysfunction may contribute to the progression of metabolic syndrome and the development of adverse outcome [22,23]. A growing body of evidence suggests that endothelial dysfunction may precede the development of insulin resistance or further promote the occurrence of manifest diabetic mellitus [24]. For example, eNOS-deficiency not only results in endothelial dysfunction, but also causes the development of insulin resistance and metabolic abnormalities, similar to those observed in diabetic mellitus [25]. The prospective investigations have also linked the increased levels of circulating markers of endothelial damage (i.e., plasminogen activator inhibitor-1 and von Willebrand factor) to the risk of incident diabetic mellitus [26].

### 1.3 Endothelial dysfunction and septic shock

Septic shock poses a serious public health problem worldwide with an overall mortality rate of 30%. Now it is recognized that a major contributor to septic morbidity and mortality is the breakdown in the function of the blood/tissue barrier due to intravascular or extra-vascular infections [27,28]. This breakdown is caused by a cascade of inflammatory events resulting in severe endothelial dysfunction which leads to systemic vascular leakage and irreversible multi-organ failure. Blood and vascular systems that respond to sepsis-associated microbial virulence factors encompass innate and adaptive immune cells, platelets, and the plasma proteins that represent antibody, complement, coagulation and fibrinolysis networks. These blood systems are enclosed by the enormous surface of the microvascular endothelium forming organ-specific vascular beds. It is vital that the blood/tissue barrier formed by this microvascular endothelium and the adjoining structures maintain their structural and functional integrity in order to support normal physiological functions of key organs. During sepsis-causing infections, the vasculature is profoundly altered by the combination of microbial virulence factors and proinflammatory mediators released from activated blood cells that gain access to surrounding tissue by crossing the leaky endothelial boundary. Severe endothelial dysfunction then results from the loss of homeostatic function of the microvascular endothelium and contributes to hypoxic injury of multiple organs. It is therefore clear that breakdown of the blood/endothelial tissue barrier is one of the major contributors to sepsis morbidity and mortality. By preventing vascular leakage through reinforcement of the endothelial barrier, it is likely that mortality from sepsis can be reduced [30,31].

#### 1.4 Endothelial dysfunction and severe virus infectious diseases

It is increasingly evident that endothelial dysfunction also contributes to the pathogenesis of a variety of potentially serious virus infectious diseases and syndromes, including dengue hemorrhagic fever, severe acute respiratory syndrome (SARS) and H1N1 influenza [32–34]. The development of severe virus infection-caused organ injury, for example, acute lung injury, has been attributed to a heightened innate immune response. Recent evidence suggests that endothelial activation, loss of barrier function, and consequent microvascular leak may also serve important mechanistic roles in the pathogenesis of severe virus infection. Shock syndrome is a dangerous complication of dengue infection and is associated with high mortality [35]. Severe dengue occurs as a result of secondary infection with a different virus serotype. Increased vascular permeability, together with myocardial dysfunction and dehydration, contribute to the development of shock, with resultant multi-organ failure [36]. The pathogenesis of shock in dengue is complex. It is known that endothelial dysfunction induced by cytokines and chemical mediators occurs. Understanding the regulatory mechanisms of endothelial dysfunction under severe virus infection may help to develop novel therapeutic strategies to cure these severe diseases.

### 2 Post-transcriptional gene regulation

Gene expression is a highly regulated process that begins with transcriptional initiation and ends with translation of a mature mRNA into protein. Between these two points, there are a series of events including processing and splicing of the pre-mRNA, export of the message from the nucleus to the cytoplasm, quality control assessment of the mRNA through the pioneer round of translation, message decay and stabilization, and translational repression and de-repression [37]. All of these events from initiation of transcription by transcription factors to the stability of the message to effective translation of the message are controlled by the presence of specific nucleotide sequences which are bound by specific RNA-binding proteins [38]. As a message is transcribed, proteins bind to form a messenger ribonucleo protein complex (mRNP) and the composition of the mRNP controls all aspects of the life of the mRNA, from pre-mRNA processing to mRNA localization to translation and degradation. Transitions between these events are accompanied by mRNP remodeling and exchange of mRNP proteins. Posttranscriptional control of gene expression, particularly mRNA stability and translation, allows for rapid changes in mRNA levels. Dysregulated mRNA stability and translation underlie a number of diseases, directly contributing to the overexpression of many genes encoding growth factors, inflammatory cytokines, and proto-oncogenes. microRNAs are an important layer that control gene expression at post-transcriptional level, which has been extensively reviewed elsewhere [9–11]. microRNAs need to function together with RNA-binding proteins. The role of RNA-binding proteins in regulation of gene expression at post-transcriptional level has been emerging.

### 3 RNA-binding proteins in vascular endothelial dysfunction

#### 3.1 Tristetraprolin

Tristetraprolin (TTP), also known as Nup475, G0S24, and TIS11, is the best known member of a class of proteins containing tandem CCCH zinc fingers. It is an mRNA-destabilizing protein that binds to AU-rich elements in labile transcripts, such as the mRNA encoding TNF, and promotes their deadenylation and degradation [39–41]. TTP destabilizes mRNA transcripts encoding multiple inflammatory modulators, including granulocyte-macrophage colony-stimulating factor (GM-CSF), IL-2, IL-6, C-FOS, iNOS, cyclooxygenase 2, CCL2, CCL3, CXC-chemokine ligand 1 (CXCL1), IFNγ and IL-10 [42–47]. As a consequence, TTP-deficient mice exhibit an early-onset, severe inflammatory phenotype, with cachexia, erosive arthritis, left-sided cardiac valvulitis, myeloid hyperplasia, and autoimmunity, which can be prevented by injection of anti-TNF antibody, or interbreeding with TNF receptor-deficient mice [48,49]. TTP promotes mRNA decay by binding directly to components of the mRNA decay machinery, including the mRNA-decapping enzymes DCP1A and DCP2, the deadenylaseCNOT6 (CCR4-NOT transcription complex subunit 6), the 5′–3′ exoribonuclease 1 (XRN1), exosome complex endonuclease PM-SCl75 (also known as RRP45) and argonaute 2 (AGO2), an argonaute protein component of the RNA-induced silencing complex [50–52]. TTP exerts its role in miRNA-dependent and independent ways [53].

It was observed that TTP-deficient mice also developed endothelial dysfunction [54]. $TTP^{-/-}$ mice showed a significant reduction of acetylcholine-induced nitric oxide-mediated vasorelaxation, which was associated with increased levels of reactive oxygen and nitrogen species. The altered reactive oxygen and nitrogen species generation correlates with increased expression of NADPH oxidase 2 resulting from enhanced NADPH oxidase 2 mRNA stability. Zhang et al. [55] recently observed that ZFP36 (TTP) is expressed in the vascular endothelium of mice with atherosclerosis but not in the vascular endothelium of normal mice.

Overexpression of TTP inhibited the expression of proinflammatory mRNA transcripts in vascular endothelial cells. The anti-inflammatory effects of TTP in endothelial cells occur via both transcriptional and post-transcriptional mechanisms. These studies suggest that enhancing vascular TTP expression might reduce vascular inflammation. Dai et al. [56] also found that TTP promoted decay of CD36 mRNA, by which it may inhibit macrophage foam-cell formation. Thus, TTP may function as an important inhibitor in the development of atherosclerosis through multiple mechanisms.

Endothelial cells are primary sensors of variations in blood oxygen concentrations. They use the hypoxia-sensitive stabilization of the hypoxia-inducible factor-1α (HIF-1α) transcription factor to engage specific transcriptional programs in response to oxygen changes. It was observed that silencing TTP in endothelial cells reverses hypoxia-induced down-regulation of HIF-1α mRNA. In addition, TTP mediated prolonged hypoxia-induced increase in the half-life of luciferase-HIF-1α-3′ UTR reporter transcript, suggesting that TTP plays a new role in the control of gene expression during the response of endothelial cell to hypoxia [57]. Besides the important role of chronic vascular inflammation, TTP is also important in regulation of acute vascular inflammation. Qiu et al. [58] recently observed that myeloid-specific TTP deficiency in mice results in extreme lipopolysaccharide sensitivity, with rapid development of typical endotoxemia signs and extensive organ damage, and elevation of serum TNF levels to 110-fold greater than that of the control. It is clear that TTP appears to regulate the different steps of mRNA processing and fate including transcription, splicing, polyadenylation, translation, and degradation. The role of TTP in vascular endothelial dysfunction is emerging. Understanding the intricacies of TTP-mediated cytokine mRNA degradation and having more detailed knowledge of the mRNAs which are regulated by TTP will identify potential targets for the development of anti-inflammatory drugs.

#### 3.2 HuR

HuR is a member of the *Drosophila* Elav protein family that binds mRNA degradation sequences and prevents RNase-mediated degradation [59,60]. HuR also binds to ARE in inflammatory cytokine mRNAs. In contrast with TTP, HuR increases the stability of tumor necrosis factor (TNF), vascular endothelial growth factor (VEGF), cyclooxygenase 2 (COX-2) and toll-like receptor 4 (TLR4) mRNAs [61–63]. HuR and TTP are both RNA-binding proteins, which are characterized as binding to the AU-rich elements (AREs) in the 3′-untranslated region (3′-UTRs) of target mRNAs. Studies have shown that some ARE-containing mRNAs are stabilized by HuR, whereas are destabilized by TTP. For example, HuR can up-regulate TNF-induced IL-6 expression by stabilizing its mRNA in human pulmonary microvascular endothelial cells, whereas TTP promotes IL-6 mRNA degradation [64]. In addition, Tiedie et al. [65] demonstrated that translation of the TNF-precursor at the ER requires expression of the ARE-binding and -stabilizing factor HuR or the absence of the ARE-binding and -destabilizing factor TTP. Phosphorylation of TTP by MK2 deceases its affinity to the ARE, inhibits its ability to replace HuR, and permits HuR-mediated initiation of translation of TNF mRNA. Activation of inflammatory pathways in the endothelium contributes to vascular diseases, including sepsis and atherosclerosis. Cheng et al. [66] demonstrated that HuR promoted endothelial activation by suppressing expression of endothelial nitric oxide synthase. Moreover, HuR also promotes the stability of ICAM-1 and VCAM-1 and increases leukocyte-endothelial cell adhesion. Knockdown of HuR with small interfering RNA (siRNA) inhibited inflammatory responses in endothelial cells, including ICAM-1 and VCAM-1 up-regulation, NF-κB phosphorylation, and adhesion of monocytes. Interestingly, tissue staining of the mouse aorta revealed increased HuR expression in the arch that is exposed to disturbed flow. These results suggest that HuR plays a critical role in inducing inflammatory response of endothelial cells under mechanical and biochemical stresses. The microvascular angiogenic response to an inflammatory stimulus was markedly diminished in the macrophages from HuR knockout mice despite the equal levels of macrophage localization to those observed in littermate wild-type controls. Furthermore, blood flow recovery and ischemic muscle neovascularization after femoral artery ligation were impaired in the conditional macrophage-specific HuR knockout mice. These results demonstrate that dynamic effects on mRNA, mediated by the RNA-binding and RNA-stabilizing protein HuR, are required for macrophage production of angiogenic factors, which play critical roles in the neovascular responses to a variety of stimuli, including tissue ischemia [67]. In another *in vitro* study, stimulation of HASMCs with LPS significantly increased the cytosolic HuR level *in vitro*. Systemic inflammation induced by LPS caused intimal hyperplasia and increased TLR4 and HuR expression. Further studies demonstrated that HuR can increase TLR4 mRNA stability by binding to its 3′UTR, which is correlated with the increased vascular smooth muscle proliferation. These results suggest that HuR contributes to regulation of hVSMC growth and homeostasis in pathologies associated with vascular smooth muscle proliferation [68]. Taken together, HuR is emerging as an important regulator in vascular biology by targeting either vascular endothelial inflammation, or VSMC proliferation or macrophage activation. It may significantly contribute to the pathogenesis of vascular inflammatory diseases such as sepsis-induced organ injury and atherosclerosis-associated diseases.

#### 3.3 MCPIP1

MCP-induced protein 1 (MCPIP1), also known as Regnase-1 orZc3h12a, was identified as a novel protein harboring a CCCH-type zinc-finger domain and a PIN-like RNase domain [69,70]. MCPIP1 mRNA expression is induced by Toll-like receptor (TLR) ligands, interleukin (IL)-1β and various stress stimuli [71–74]. MCPIP1 functions as an important negative regulator in both adaptive and innate immune response through destabilizing mRNAs encoding immune related proteins including IL-6, IL-2 and IL-12p40 via their 3′ untranslated regions [71,72,75]. As a consequence, Mcpip1-deficient mice developed severe systemic inflammation, characterized by growth retardation, splenomegaly, lymphadenopathy, severe anemia and premature death [71,76]. In addition, the serum levels of proinflammatory cytokine and production of autoantibodies are also dramatically increased in Mcpip1-deficient mice [77]. MCPIP1 was firstly identified as an endogenous inhibitor in macrophage-induced inflammation. Under normal condition, MCPIP1 protein is highly enriched in the mouse lung, spleen, thymus, colon and intestine. MCPIP1 expression was significantly induced in macrophages by bacterium and virus infection and by endogenous cytokines such as TNFα and IL-1β. Overexpression of MCPIP1 in macrophages significantly suppressed the proinflammatory cytokine production such as IL-6 and IL-12. In the Mcpip1-deficient macrophages, the expression of IL-6 was increased as its mRNA decay was impaired [71,72]. Besides macrophages, our previous studies also showed that MCPIP1 expression is up-regulated in the endothelial cells and VSMCs in the advanced atherosclerotic lesions. In culturing human umbilical vein endothelial cells (HUVECs), MCPIP1 expression was significantly induced by inflammatory cytokines TNFα and IL-1β and overexpression of MCPIP1 suppresses cytokine-induced expression of VCAM-1, as well as monocyte adhesion to human ECs [78]. These studies demonstrated MCPIP1 as a feedback control of cytokine-induced endothelial inflammation and suggest that MCPIP1 may be a critical regulator in vascular diseases such as sepsis and atherosclerosis. Indeed, Mcpip1-deficient mice are extremely sensitive to LPS-induced septic shock [79]. Especially, a minimum LPS challenge can cause severe inflammatory lung and liver injury and death in Mcpip1-deficient mice, suggesting that Mcpip1 may play an important role in maintaining the vascular homeostasis under severe bacterium and virus infection. Activator of MCPIP1 enzymatic action may have therapeutic benefits for the patients with sepsis, severe viral infection and atherosclerosis-associated diseases. Besides MCPIP1, the other member in MCPIP1 protein family Zc3h12c (also known as MCPIP3) also significantly inhibited the endothelial cell inflammatory response *in vitro*. Overexpression of Zc3h12c significantly attenuated TNFα-induced expression of chemokines and adhesive molecules, and thus reduced monocyte adherence to HUVECs. Conversely, siRNA-mediated knockdown of Zc3h12c increased the TNFα-induced expression of chemokines and adhesive molecules in HUVECs. Furthermore, forced expression of Zc3h12c decreased TNFα-induced IKKα/β (IκB (inhibitor of nuclear factor κB) kinase α/β), IκBα phosphorylation and p65 nuclear translocation, suggesting that Zc3h12c exerted its anti-inflammatory function probably by suppressing the NF-κB (nuclear factor κB) pathway. Thus Zc3h12c is also an endogenous inhibitor of TNFα-induced inflammatory signaling in HUVECs and might be a therapeutic target in vascular inflammatory diseases [80]. How does MCPIP1 selectively target some specific mRNAs? Structural and biochemical analysis demonstrated that MCPIP1 contains a PIN-like RNase domain and can directly degrade mRNA *in vitro* [81]. In the cells, MCPIP1 is predominantly localized in cytoplasm as small granule-like pattern. We have found that MCPIP1 is co-localized with GW-182 and Ago-2, which are major components of miRNA-mediated RNA silencing complex [82], suggesting that MCPIP1 may be involved in the miRNA-effector pathway. Further studies are needed to clarify this mechanism. Interestingly, Suzuki et al. [83] reported that MCPIP1 can specifically recognize the terminal loops of precursors miRNAs and suppress miRNA biosynthesis, suggesting that MCPIP1 may affect cell behavior by influencing the miRNA generation.

#### 3.4 Drosha and DGCR8

microRNAs (miRNAs) represent a family of conserved short (≈22 nt) noncoding single-strand RNAs that have been identified in plants and animals. They are generated by the sequential processing of the RNA template by the enzyme Drosha and Dicer, and mature miRNAs can regulate the levels of gene expression at the posttranscriptional level. microRNAs participate in a diverse range of regulatory events via regulation of genes involved in the control of process such as development, differentiation, homeostasis, metabolism, growth, proliferation, and apoptosis [9–11]. microRNAs are highly expressed in endothelial cells and they regulate various aspects of vascular endothelial biology, which have been extensively reviewed elsewhere [12–15]. In this review, we focus on the miRNA biogenesis enzyme Drosha and Dicer as well as RNA-binding protein DGCR8. They not only function through generation of miRNAs but also function independently of miRNAs. Drosha cleaves double-stranded primary miRNA by interacting with double-stranded RNA binding protein DGCR8 and processes primary miRNA into precursor miRNA to participate in the miRNA biogenesis pathway. Fan et al. [84] found that disruption of Drosha in VSMCs resulted in embryonic lethality at E14.5 with severe liver hemorrhage in mutant embryos. The vascular structure was absent in the yolk sac of Drosha homozygotes at E14.5. Loss of Drosha reduced VSMC proliferation *in vitro* and *in vivo*. The VSMC differentiation marker genes, including αSMA, SM22, and CNN1, and endothelial cell marker CD31 were significantly down-regulated in Drosha conditional knockout (CKO) mice compared to controls. ERK1/2 mitogen-activated protein kinase and the phosphatidylinositol 3-kinase/AKT were attenuated in VSMCs *in vitro* and *in vivo*. These data demonstrated that Drosha is required for VSMC survival by targeting multiple signaling pathways [84]. Consistently, Chen et al. [85] also found that loss of DGCR8 in VSMCs resulted in extensive liver hemorrhage and embryonic mortality between embryonic days (E) 12.5 and E13.5. DGCR8 CKO embryos displayed dilated blood vessels and disarrayed vascular architecture. Blood vessels were absent in the yolk sac ofDGCR8 KOs after E12.5. Disruption of DGCR8 in VSMCs reduced VSMC proliferation and promoted apoptosis *in vitro* and *in vivo*. In DGCR8 CKO embryos and knockout VSMCs, differentiation marker genes, including αSMA, SM22, and CNN1, were significantly down-regulated, and the survival pathways of ERK1/2 mitogen-activated protein kinase and the phosphatidylinositol 3-kinase/AKT were attenuated. The mechanisms that cause these phenotypes are not completely clear. Knockout of DGCR8 in VSMCs has led to down-regulation of the miR-17/92 and miR-143/145 clusters, suggesting that the misprocessing of miRNA maturation may contribute to the abnormal differentiation and proliferation of VSMCs [85]. Pan et al. [86] also reported that conditionally deleted the miRNA-processing enzyme Dicer in the proepicardium using Gata5-Cre mice leads to impaired epicardial epithelial-to-mesenchymal transition and reduction in epicardial cell proliferation and differentiation into coronary smooth muscle cells.

#### 3.5 Argonaute 2

Argonaute 2 (Ago2) is a central component of RNA-induced silencing complex and plays a key role in RNA interference and miRNA effector pathway. Ago2 is expressed in vascular endothelial cells and may play an essential role in regulation of endothelial dysfunction in miRNA-dependent mechanisms. In addition, some reported that Ago2, as a RNA binding protein, may function in a miRNA-independent way. Asai et al. [87] reported that knocking down of Ago2 significantly suppressed VEGF- induced angiogenesis, which suggests that Ago2 is required for angiogenesis. Endothelial cell migration induced in response to VEGF is a crucial step of angiogenesis and it is dependent on the activation of the p38 MAP-kinase pathway downstream of VEGFR2. Pin et al. [88] found that overexpression of Ago2 impaired VEGF-induced p38 activation and endothelial cell migration. The role of Ago2 in endothelial dysfunction needs to be further explored.

#### 3.6 Others

The RNA-binding protein Quaking (QKI) is a member of the “STAR” (signal transduction and activation of RNA) family. Proteins in this family are characterized by the presence of RNA-binding motif, KH domains, as well as SH2 and SH3 domains and potential phosphorylation sites, suggesting that they function in signal transduction pathways [89]. It is well known that QKI plays an important role in the postnatal central nervous system during myelination. Recent studies suggest that it also plays an essential role in blood vessel development [90]. In addition, van der Veer et al. [91] observed that QKI is highly expressed in neointimal VSMCs of human coronary restenoticlesions and neointima hyperplasia of mice. Abrogation of QKI attenuated fibroproliferative properties of VSMC and potently induced contractile apparatus protein expression. Further studies indicate that QKI localizes to the spliceosome and regulates myocardin splicing. Similarly, RA301/Trabeta, a sequence-specific RNA-binding protein, has also been found highly expressed in coronary artery with intimal thickening, and atherosclerotic aorta. RA301/Trabeta seems to also regulate VSMC proliferation [92]. The roles of these RNA-binding proteins in vascular endothelial dysfunction need to be further explored.

### 4 Conclusion and future direction

Normal EC function is critical for all aspects of vascular

**Table 1** Overview of RNA-binding proteins and their function

| RNA-binding proteins | Function | Refs |
|---|---|---|
| Tristetraprolin | Inhibition of vascular inflammation by promoting mRNA degradation of inflammatory cytokines | [39–41,42–47,55,56] |
| HuR | Promotion of endothelial activation by stabilizing the mRNAs of cytokines and adhesion molecules | [61–63,66] |
| MCPIP1 | Inhibition of vascular inflammation by degradation the mRNA of inflammatory cytokines and suppressing NF-κB signaling | [69–74,78,79] |
| Zc3h12c | Inhibition of vascular inflammation by suppressing NF-κB signaling | [80] |
| Drosha | Required for VSMC survival | [84] |
| DGCR8 | Required for VSMC differentiation and proliferation | [85] |
| Argonaute 2 | Promotion of VEGF-induced angiogenesis | [87] |
| Quaking | Promotion of fibroproliferative properties of VSMC | [88] |
| RA301/Trabeta | Regulation of VSMC proliferation | [89] |

homeostasis, such as control of blood vessel development, growth and differentiation; control of leukocyte trafficking; control of vascular tone; control of vascular barrier; control of platelet function, coagulation and fibrinolysis. Vascular endothelial dysfunction is associated with many important diseases such as atherosclerosis-associated heart attack and stroke, diabetes, sepsis and septic shock, severe virus infection-caused organ injury. Understanding of the pathogenesis and molecular regulatory mechanisms will provide insights into development of new therapeutic approaches to cure these diseases. microRNAs represent a novel layer that controls the gene expression at a post-transcriptional level and have emerged as important regulators in endothelial dysfunction. However, miRNAs do not function alone. microRNAs must get the jobs done by closely cooperating with RNA-binding proteins. Increasing evidences suggest that the RNA-binding proteins such as TTP, MCPIP1, HuR, Drosha, and DGCR8 act as important components in gene expression at post-transcriptional level in a miRNA-dependent and/or independent manner. Their roles in regulation of vascular endothelial dysfunction are also characterized in recent publications (Table 1). Further studies *in vivo* and translational to human diseased samples will be definitely needed.

*This work was supported by the National Natural Science Foundation of China (91339113, 81270202, 81070095 to Xin HongBo) and the National Basic Research Program of China (2013CB531103 to Xin HongBo).*

1 Rajendran P, Rengarajan T, Thangavel J, Nishigaki Y, Sakthisekaran D, Sethi G,Nishigaki I. The vascular endothelium and human diseases. Int J Biol Sci, 2013, 9: 1057–1069
2 Cheng Z, Yang X, Wang H. Hyperhomocysteinemia and Endothelial Dysfunction. CurrHypertens Rev, 2009, 5: 158–165
3 Herrera MD, Mingorance C, Rodríguez-Rodríguez R, Alvarez de Sotomayor M. Endothelial dysfunction and aging: an update. Ageing Res Rev, 2010, 9: 142–152
4 Winn RK, Harlan JM. The role of endothelial cell apoptosis in inflammatory and immune diseases. J ThrombHaemost, 2005, 3: 1815–1824
5 Sahni SK. Endothelial cell infection and hemostasis. Thromb Res, 2007, 119: 531–549
6 Menghini R, Casagrande V, Federici M. microRNAs in endothelial senescence and atherosclerosis. J CardiovascTransl Res, 2013, 6: 924–930
7 Anderson P. Post-transcriptional regulons coordinate the initiation and resolution of inflammation. Nat Rev Immunol, 2010, 10: 24–35
8 Anderson P. Post-transcriptional control of cytokine production. Nat Immunol, 2009, 9: 353–359
9 Small EM, Olson EN. Pervasive roles of microRNAs in cardiovascular biology. Nature, 2011, 469: 336–342
10 Ambros V. The functions of animal microRNAs. Nature, 2004, 431: 350–355
11 Cullen BR. Viral and cellular messenger RNA targets of viral microRNAs. Nature, 2009, 457: 421–425
12 Condorelli G, Latronico MV, Cavarretta E. microRNAs in cardiovascular diseases: current knowledge and the road ahead. J Am CollCardiol, 2014, pii: S0735-1097(14)01108-5
13 Chamorro-Jorganes A, Araldi E, Suárez Y. microRNAs as pharmacological targets in endothelial cell function and dysfunction. Pharmacol Res, 2013, 75: 15–27
14 Madrigal-Matute J, Rotllan N, Aranda JF, Fernández-Hernando C. microRNAs and atherosclerosis. Curr Atheroscler Rep, 2013, 15: 322
15 Santoro MM, Nicoli S. miRNAs in endothelial cell signaling: the endomiRNAs. Exp Cell Res, 2013, 319: 1324–1330
16 Gresele P, Falcinelli E, Sebastiano M, Baldelli F. Endothelial and platelet function alterations in HIV-infected patients. Thromb Res, 2012, 129: 301–308
17 Charreau B. Molecular regulation of endothelial cell activation: novel mechanisms and emerging targets. Curr Opin Organ Transplant, 2011, 16: 207–213
18 Lusis AJ. Atherosclerosis. Nature, 2000, 407: 233–241
19 Libby P. Inflammation in atherosclerosis. Nature, 2002, 420: 868–874
20 Polovina MM, Potpara TS. Endothelial dysfunction in metabolic and vascular disorders. Postgrad Med, 2014, 126: 38–53
21 Gutiérrez E, Flammer AJ, Lerman LO, Elízaga J, Lerman A, Fernández-Avilés F. Endothelial dysfunction over the course of coronary artery disease. Eur Heart J, 2013, 34: 3175–3181
22 Sena CM, Pereira AM, Seiça R. Endothelial dysfunction - a major mediator of diabetic vascular disease. Biochim Biophys Acta, 2013, 1832: 2216–2231
23 Barton M. Prevention and endothelial therapy of coronary artery disease. Curr Opin Pharmacol, 2013, 13: 226–241
24 Mauricio MD, Aldasoro M, Ortega J, Vila JM. Endothelial dysfunction in morbid obesity. Curr Pharm Des, 2013, 19: 5718–5729
25 Picchi A, Gao X, Belmadani S, Potter BJ, Focardi M, Chilian WM, Zhang C. Tumor necrosis factor-alpha induces endothelial dysfunction in the prediabetic metabolic syndrome. Circ Res, 2006, 99: 69–77
26 Imamura A, Takahashi R, Murakami R, Kataoka H, Cheng XW, Numaguchi Y, MuroharaT, Okumura K. The effects of endothelial nitric oxide synthase gene polymorphisms on endothelial function and metabolic risk factors in healthy subjects: the significance of plasma adiponectin levels. Eur J Endocrinol, 2008, 158: 189–195
27 Deutschman CS, Tracey KJ. Sepsis: current dogma and new perspectives. Immunity, 2014, 40: 463–475
28 De Backer D, Orbegozo Cortes D, Donadello K, Vincent JL. Pathophysiology of microcirculatory dysfunction and the pathogenesis of septic shock. Virulence, 2014, 5: 73–79
29 Müller MM, Griesmacher A. Markers of endothelial dysfunction. ClinChem Lab Med, 2000, 38: 77–85
30 Page AV, Liles WC. Biomarkers of endothelial activation/dysfunction in infectious diseases. Virulence, 2013, 4: 507–516
31 Lee WL, Liles WC. Endothelial activation, dysfunction and permeability during severe infections. Curr Opin Hematol, 2011, 18: 191–196
32 Armstrong SM, Darwish I, Lee WL. Endothelial activation and dysfunction in the pathogenesis of influenza A virus infection. Virulence, 2013, 4: 537–542
33 Gresele P, Falcinelli E, Sebastiano M, Baldelli F. Endothelial and platelet function alterations in HIV-infected patients. Thromb Res, 2012, 129: 301–308
34 Charreau B. Molecular regulation of endothelial cell activation: novel mechanisms and emerging targets. Curr Opin Organ Transplant, 2011, 16: 207–213
35 Liu P, Woda M, Ennis FA, Libraty DH. Dengue virus infection differentially regulates endothelial barrier function over time through type I interferon effects. J Infect Dis, 2009, 200: 191–201
36 Rajapakse S. Dengue shock. J Emerg Trauma Shock, 2011, 4: 120–127
37 Whelan JT, Hollis SE, Cha DS, Asch AS, Lee MH. Post-transcriptional regulation of the Ras-ERK/MAPK signaling pathway. J Cell Physiol, 2012, 227: 1235–1241
38 Glisovic T, Bachorik JL, Yong J, Dreyfuss G. RNA-binding proteins and post-transcriptional gene regulation. FEBS Lett, 2008, 582: 1977–1986
39 Brooks SA, Blackshear PJ. Tristetraprolin (TTP): interactions with mRNA and proteins, and current thoughts on mechanisms of action. Biochim Biophys Acta, 2013, 1829: 666–679

40 Ciais D, Cherradi N, Feige JJ. Multiple functions of tristetraprolin/TIS11 RNA-binding proteins in the regulation of mRNA biogenesis and degradation. Cell Mol Life Sci, 2013, 70: 2031–2044

41 Carrick DM, Lai WS, Blackshear PJ. The tandem CCCH zinc finger protein tristetraprolin and its relevance to cytokine mRNA turnover and arthritis. Arthritis Res Ther, 2004, 6: 248–264

42 Carballo E, Lai WS, Blackshear PJ. Evidence that tristetraprolin is a physiological regulator of granulocyte-macrophage colony-stimulating factor messenger RNA deadenylation and stability. Blood, 2000, 95: 1891–1899

43 Ogilvie RL, Abelson M, Hau HH, Vlasova I, Blackshear PJ, Bohjanen PR. Tristetraprolin down-regulates IL-2 gene expression through AU-rich element-mediated mRNA decay. J Immunol, 2005, 174: 953–961

44 Zhao W, Liu M, D'Silva NJ, Kirkwood KL. Tristetraprolin regulates interleukin-6 expression through p38 MAPK-dependent affinity changes with mRNA 3′ untranslated region. J Interferon Cytokine Res, 2011, 31: 629–637

45 Fechir M, Linker K, Pautz A, Hubrich T, Förstermann U, Rodriguez-Pascual F, Kleinert H. Tristetraprolin regulates the expression of the human inducible nitric-oxide synthase gene. Mol Pharmacol, 2005, 67: 2148–2161

46 Datta S, Biswas R, Novotny M, Pavicic PG Jr, Herjan T, Mandal P, Hamilton TA. Tristetraprolin regulates CXCL1 (KC) mRNA stability. J Immunol, 2008, 180: 2545–2552

47 Stoecklin G, Tenenbaum SA, Mayo T, Chittur SV, George AD, Baroni TE, Blackshear PJ, Anderson P. Genome-wide analysis identifies interleukin-10 mRNA as target of tristetraprolin. J Biol Chem, 2008, 283: 11689–11699

48 Taylor GA, Carballo E, Lee DM, Lai WS, Thompson MJ, Patel DD, Schenkman DI, Gilkeson GS, Broxmeyer HE, Haynes BF, Blackshear PJ. A pathogenetic role for TNFalpha in the syndrome of cachexia, arthritis, and autoimmunity resulting from tristetraprolin (TTP) deficiency. Immunity, 1996, 4: 445–454

49 Carballo E, Lai WS, Blackshear PJ. Feedback inhibition of macrophage tumor necrosis factor-alpha production by tristetraprolin. Science, 1998, 281: 1001–1005

50 Blackshear PJ, Perera L. Phylogenetic Distribution and evolution of the linked RNA-binding and NOT1-binding domains in the tristetraprolin family of tandem CCCH zinc finger proteins. J Interferon Cytokine Res, 2014, 34: 297–306

51 Lai WS, Perera L, Hicks SN, Blackshear PJ. Mutational and structural analysis of the tandem zinc finger domain of tristetraprolin. J Biol Chem, 2014, 289: 565–580

52 Fabian MR, Frank F, Rouya C, Siddiqui N, Lai WS, Karetnikov A, Blackshear PJ, Nagar B, Sonenberg N. Structural basis for the recruitment of the human CCR4-NOT deadenylase complex by tristetraprolin. Nat StructMol Biol, 2013, 20: 735–739

53 Jing Q, Huang S, Guth S, Zarubin T, MotoyamaA, Chen J, Di Padova F, Lin SC, Gram H, Han J. Involvement of microRNA in AU-rich element-mediated mRNA instability. Cell, 2005, 120: 623–634

54 Bollmann F, Wu Z, Oelze M, Siuda D, Xia N, Henke J, Daiber A, Li H, Stumpo DJ, Blackshear PJ, Kleinert H, Pautz A. Endothelial dysfunction in tristetraprolin-deficient mice is not caused by enhanced TNF-α expression. J Biol Chem, 2014, 289: 15653–15665

55 Zhang H, Taylor WR, Joseph G, Caracciolo V, Gonzales DM, Sidell N, Seli E, Blackshear PJ, Kallen CB. mRNA-binding protein ZFP36 is expressed in atherosclerotic lesions and reduces inflammation in aortic endothelial cells. Arterioscler Thromb Vasc Biol, 2013, 33: 1212–1220

56 Dai XY, Cai Y, Sun W, Ding Y, Wang W, Kong W, Tang C, Zhu Y, Xu MJ, Wang X. Intermedin inhibits macrophage foam-cell formation via tristetraprolin-mediated decay of CD36 mRNA. Cardiovasc Res, 2014, 101: 297–305

57 Chamboredon S, Ciais D, Desroches-Castan A, Savi P, Bono F, Feige JJ, CherradiN. Hypoxia-inducible factor-1α mRNA: a new target for destabilization by tristetraprolin in endothelial cells. MolBiol Cell, 2011, 22: 3366–3378

58 Qiu LQ, Stumpo DJ, Blackshear PJ. Myeloid-specific tristetraprolin deficiency in mice results in extreme lipopolysaccharide sensitivity in an otherwise minimal phenotype. J Immunol, 2012, 188: 5150–5159

59 Chang SH, Hla T. Post-transcriptional gene regulation by HuR and microRNAs in angiogenesis. Curr Opin Hematol, 2014, 21: 235–240

60 Pullmann R Jr, Rabb H. HuR and other turnover- and translation-regulatory RNA-binding proteins: implications for the kidney. Am J Physiol Renal Physiol, 2014, 306: F569–576

61 Ceolotto G, de Kreutzenberg SV, Cattelan A, Fabricio AS, Squarcina E, Gion M, Semplicini A, Fadini GP, Avogaro A. Sirtuin 1 stabilization by HuR represses TNF-α and glucose induced E-selectin release and endothelial cell adhesiveness *in vitro*. Relevance to human metabolic syndrome. Clin Sci (Lond), 2014, 127: 449–461

62 Kurosu T, Ohga N, Hida Y, Maishi N, Akiyama K, Kakuguchi W, Kuroshima T, Kondo M, Akino T, Totsuka Y, Shindoh M, Higashino F, Hida K. HuR keeps an angiogenic switch on by stabilising mRNA of VEGF and COX-2 in tumour endothelium. Br J Cancer, 2011, 104: 819–829

63 Lin FY, Chen YH, Lin YW, Tsai JS, Chen JW, Wang HJ, Chen YL, Li CY, Lin SJ. The role of human antigen R, an RNA-binding protein, in mediating the stabilization of toll-like receptor 4 mRNA induced by endotoxin: a novel mechanism involved in vascular inflammation. Arterioscler Thromb Vasc Biol, 2006, 26: 2622–2629

64 Shi JX, Su X, Xu J, Zhang WY, Shi Y. HuR post-transcriptionally regulates TNF-α-induced IL-6 expression in human pulmonary microvascular endothelial cells mainly via tristetraprolin. Respir Physiol Neurobiol, 2012, 181: 154–161

65 Tiedje C, Ronkina N, Tehrani M, Dhamija S, Laass K, Holtmann H, Kotlyarov A, Gaestel M. The p38/MK2-driven exchange between tristetraprolin and HuR regulates AU-rich element-dependent translation. PLoS Genet, 2012, 8: e1002977

66 Cheng HS, Sivachandran N, Lau A, Boudreau E, Zhao JL, Baltimore D, Delgado-Olguin P, Cybulsky MI, Fish JE. microRNA-146 represses endothelial activation by inhibiting pro-inflammatory pathways. EMBO Mol Med, 2013, 5: 949–966

67 Zhang J, Modi Y, Yarovinsky T, Yu J, Collinge M, Kyriakides T, Zhu Y, SessaWC, Pardi R, Bender JR. Macrophage β2 integrin-mediated, HuR-dependent stabilization of angiogenic factor-encoding mRNAs in inflammatory angiogenesis. Am J Pathol, 2012, 180: 1751–1760

68 Pullmann R Jr, Juhaszova M, López de Silanes I, Kawai T, Mazan-Mamczarz K, Halushka MK, Gorospe M. Enhanced proliferation of cultured human vascular smooth muscle cells linked to increased function of RNA-binding protein HuR. J Biol Chem, 2005, 280: 22819–22826

69 Uehata T, Akira S. mRNA degradation by the endoribonuclease-eRegnase-1/ZC3H12a/MCPIP-1. Biochim Biophys Acta, 2013, 1829: 708–713

70 Jura J, Skalniak L, Koj A. Monocyte chemotactic protein-1-induced protein-1(MCPIP1) is a novel multifunctional modulator of inflammatory reactions. Biochim Biophys Acta, 2012, 1823: 1905–1913

71 Matsushita K, Takeuchi O, Standley DM, Kumagai Y, Kawagoe T, Miyake T, Satoh T, Kato H, Tsujimura T, Nakamura H, Akira S. Zc3h12a is an RNase essential for controlling immune responses by regulating mRNA decay. Nature, 2009, 458: 1185–1190

72 Liang J, Wang J, Azfer A, Song W, Tromp G, Kolattukudy PE, Fu M. A novel CCCH-zinc finger protein family regulates proinflammatory activation of macrophages. J Biol Chem, 2008, 283: 6337–6346

73 Liang J, Song W, Tromp G, Kolattukudy PE, Fu M. Genome-wide survey and expression profiling of CCCH-zinc finger family reveals a functional module inmacrophage activation. PLoS ONE, 2008, 3: e2880

74 Mizgalska D, Wegrzyn P, Murzyn K, Kasza A, Koj A, Jura J, Jarzab B, Jura J. Interleukin-1-inducible MCPIP protein has structural and functional properties of RNase and participates in degradation of IL-1beta mRNA. FEBS J, 2009, 276: 7386–7399

75 Li M, Cao W, Liu H, Zhang W, Liu X, Cai Z, GuoJ, Wang X, Hui Z, Zhang H, Wang J, Wang L. MCPIP1 down-regulates IL-2 expression through an ARE-independent pathway. PLoS ONE, 2012, 7: e49841

76 Liang J, Saad Y, Lei T, Wang J, Qi D, Yang Q, Kolattukudy PE, Fu

M. MCP-induced protein 1 deubiquitinates TRAF proteins and negatively regulates JNK and NF-kappaB signaling. J Exp Med, 2010, 207: 2959–2973
77 Miao R, Huang S, Zhou Z, Quinn T, Van Treeck B, Nayyar T, Dim D, Jiang Z, Papasian CJ, Eugene Chen Y, Liu G, Fu M. Targeted disruption of MCPIP1/Zc3h12a results in fatal inflammatory disease. Immunol Cell Biol, 2013, 91: 368–376
78 Qi Y, Liang J, She ZG, Cai Y, Wang J, Lei T,Stallcup WB, Fu M. MCP-induced protein 1 suppresses TNFalpha-induced VCAM-1 expression in human endothelial cells. FEBS Lett, 2010, 584: 3065–3072
79 Huang S, Miao R, Zhou Z, Wang T, Liu J, Liu G, Chen YE, Xin HB, Zhang J, Fu M. MCPIP1 negatively regulates toll-like receptor 4 signaling and protects mice from LPS-induced septic shock. Cell Signal, 2013, 25: 1228–1234
80 Liu L, Zhou Z, Huang S, Guo Y, Fan Y, Zhang J, Zhang J, Fu M, Chen YE. Zc3h12c inhibits vascular inflammation by repressing NF-κB activation and pro-inflammatory gene expression in endothelial cells. Biochem J, 2013, 451: 55–60
81 Xu J, Peng W, Sun Y, Wang X, Xu Y, Li X, Gao G, Rao Z. Structural study of MCPIP1 N-terminal conserved domain reveals a PIN-like RNase. Nucleic Acids Res, 2012, 40: 6957–6965
82 Qi D, Huang S, Miao R, She ZG, Quinn T, Chang Y, Liu J, Fan D, Chen YE, Fu M. Monocyte chemotactic protein-induced protein 1 (MCPIP1) suppresses stress granule formation and determines apoptosis under stress. J Biol Chem, 2011, 286: 41692–41700
83 Suzuki HI, Arase M, Matsuyama H, Choi YL, Ueno T, Mano H, Sugimoto K, MiyazonoK. MCPIP1 ribonuclease antagonizes dicer and terminates microRNA biogenesis through precursor microRNA degradation. Mol Cell, 2011, 44: 424–436
84 Fan P, Chen Z, Tian P, Liu W, Jiao Y, Xue Y, Bhattacharya A, Wu J, Lu M, GuoY, Cui Y, Gu W, Gu W, Yue J. miRNA biogenesis enzyme Drosha is required for vascular smooth muscle cell survival. PLoS ONE, 2013, 8: e60888
85 Chen Z, Wu J, Yang C, Fan P, Balazs L, Jiao Y, Lu M, Gu W, Li C, Pfeffer LM, Tigyi G, Yue J. DiGeorge syndrome critical region 8 (DGCR8) protein-mediated microRNA biogenesis is essential for vascular smooth muscle cell development in mice. J Biol Chem, 2012, 287: 19018–19028
86 Pan Y, Balazs L, Tigyi G, Yue J. Conditional deletion of Dicer in vascular smooth muscle cells leads to the developmental delay and embryonic mortality. Biochem Biophys Res Commun, 2011, 408: 369–374
87 Asai T, Suzuki Y, Matsushita S, Yonezawa S, Yokota J, Katanasaka Y, Ishida T, Dewa T, Kiwada H, Nango M, Oku N. Disappearance of the angiogenic potential of endothelial cells caused by Argonaute 2 knockdown. Biochem Biophys Res Commun, 2008, 368: 243–248
88 Pin AL, Houle F, Guillonneau M, Paquet ER, Simard MJ, Huot J. miR-20a represses endothelial cell migration by targeting MKK3 and inhibiting p38 MAP kinase activation in response to VEGF. Angiogenesis, 2012, 15: 593–608
89 Justice MJ, Hirschi KK. The role of quaking in mammalian embryonic development. AdvExp Med Biol, 2010, 693: 82–92
90 van der Veer EP, de Bruin RG, Kraaijeveld AO, de Vries MR, Bot I, Pera T, Segers FM, Trompet S, van Gils JM, Roeten MK, Beckers CM, van Santbrink PJ, Janssen A, van Solingen C, Swildens J, de Boer HC, Peters EA, Bijkerk R, Rousch M, Doop M, Kuiper J, Schalij MJ, van der Wal AC, Richard S, van Berkel TJ, Pickering JG, Hiemstra PS, Goumans MJ, Rabelink TJ, de Vries AA, Quax PH, Jukema JW, Biessen EA, van Zonneveld AJ. Quaking, an RNA-binding protein, is a critical regulator of vascular smooth muscle cell phenotype. Circ Res, 2013, 113: 1065–1075
91 Noveroske JK, Lai L, Gaussin V, Northrop JL, Nakamura H, Hirschi KK, Justice MJ. Quaking is essential for blood vessel development. Genesis, 2002, 32: 218–230
92 Tsukamoto Y, Matsuo N, Ozawa K, Hori O, Higashi T, Nishizaki J, Tohnai N, Nagata I, Kawano K, Yutani C, Hirota S, Kitamura Y, Stern DM, Ogawa S. Expression of a novel RNA-splicing factor, RA301/Tra2beta, in vascular lesions and its role in smooth muscle cell proliferation. Am J Pathol, 2001, 158: 1685–1694

**Open Access** This article is distributed under the terms of the Creative Commons Attribution License which permits any use, distribution, and reproduction in any medium, provided the original author(s) and source are credited.
